# Contact-Chemosensory Evolution Underlying Reproductive Isolation in *Drosophila* Species

**DOI:** 10.3389/fnbeh.2020.597428

**Published:** 2020-12-04

**Authors:** Kosei Sato, Daisuke Yamamoto

**Affiliations:** Neuro-Network Evolution Project, Advanced ICT Research Institute, National Institute of Information and Communications Technology, Kobe, Japan

**Keywords:** premating isolation, pheromones, hybrids, hydrocarbon metabolism, gustatory receptors, central integration, fruitless, doublesex

## Abstract

The main theme of the review is how changes in pheromone biochemistry and the sensory circuits underlying pheromone detection contribute to mate choice and reproductive isolation. The review focuses primarily on gustatory and non-volatile signals in *Drosophila*. Premating isolation is prevalent among closely related species. In *Drosophila*, preference for conspecifics against other species in mate choice underlies premating isolation, and such preference relies on contact chemosensory communications between a female and male along with other biological factors. For example, although *D. simulans* and *D. melanogaster* are sibling species that yield hybrids, their premating isolation is maintained primarily by the contrasting effects of 7,11-heptacosadiene (7,11-HD), a predominant female pheromone in *D. melanogaster*, on males of the two species: it attracts *D. melanogaster* males and repels *D. simulans* males. The contrasting preference for 7,11-HD in males of these two species is mainly ascribed to opposite effects of 7,11-HD on neural activities in the courtship decision-making neurons in the male brain: 7,11-HD provokes both excitatory and inhibitory inputs in these neurons and differences in the balance between the two counteracting inputs result in the contrasting preference for 7,11-HD, i.e., attraction in *D. melanogaster* and repulsion in *D. simulans*. Introduction of two double bonds is a key step in 7,11-HD biosynthesis and is mediated by the desaturase desatF, which is active in *D. melanogaster* females but transcriptionally inactivated in *D. simulans* females. Thus, 7,11-HD biosynthesis diversified in females and 7,11-HD perception diversified in males, yet it remains elusive how concordance of the changes in the two sexes was attained in evolution.

## Introduction

The lack of gene flow or reproductive isolation is a prerequisite for the persistence of any species inhabiting the same place (Coyne and Orr, 2004). Premating as well as postmating isolation play roles in interfering with free gene flow, although neither would work as a perfect barrier against “interspecific hybridization” between populations that recently diverged. There exist cases where two populations of animals can produce fertile offspring and thus are judged to belong to the same species, yet mating between two individuals each from an alternative population barely happens in nature, implying that premating isolation could precede the development of postmating isolation (Shumer et al., 2017). The African and cosmopolitan populations of *Drosophila melanogaster* undergo such an incipient speciation that was driven by premating isolation (Wu et al., 1995). Conversely, postmating isolation may occur prior to the development of premating isolation (Sweigart, 2010): the interspecific crosses happen at a high rate between *D. virilis* and *D. americana*, yet fertilization of eggs after mating hardly occurs in such crosses. In contrast to postmating isolation, premating isolation inevitably requires some cognitive process for discriminating a conspecific candidate partner from individuals of closely related species. If premating isolation takes place under the conditions where interspecies hybrids do not suffer from discernible fertility decrement (as expected to be the case for incipient speciation), assortative mating would likely be favored by sexual selection even when the adaptive (or fitness) advantage is limited. Here questions arise as to how the “perceptual shift” to favor a particular sexual trait in a potential mate develops and what genetic and neural mechanisms underlie this shift. *Drosophila* flies offer an ideal platform for addressing these evolutionary questions because of the comprehensive resource for genetic and neurobiological analyses in the model species *D. melanogaster* and because of the rich collection of species in the *Drosophila* phylogeny exhibiting distinct anatomical and behavioral characteristics (Hales et al., 2015).

This review covers mechanistic aspects of mating behavior, because the mechanistic understanding is critical for deciphering how animal behavior diversified thorough evolution. Homologous circuits that underly homologous behaviors need to be compared across species at the level of single cells, in which genes involved in behavioral divergence exert their specific actions. We review the current understanding of contact chemosensory mechanisms by which flies recognize conspecifics and discuss how species-specificity in pheromone perception and mate preference diversified in evolution.

## Pheromone Production

**FIGURE 1:**
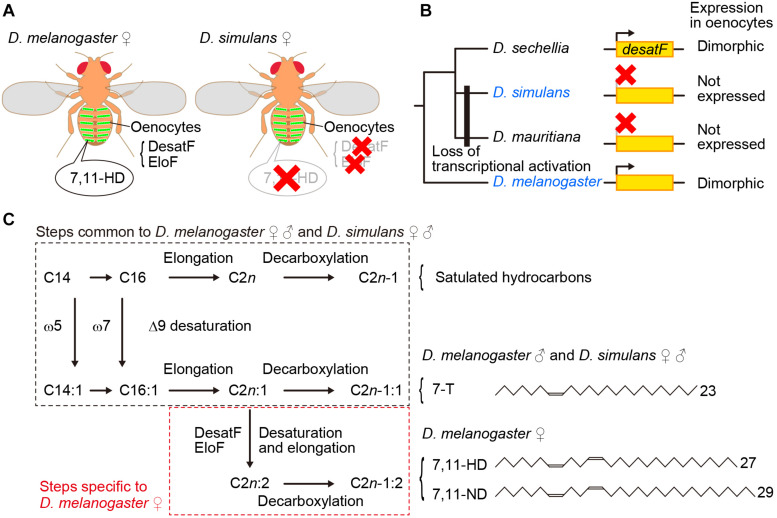
7,11-HD plays a key role for male mate choice in the *D. melanogaster* species subgroup. **(A)** 7,11-HD is a *D. melanogaster* female-specific pheromone synthesized in oenocytes as mediated by enzymes including DesatF and EloF. **(B)** DesatF expression is female-specific in *D. melanogaster* and *D. sechellia*, whereas it is transcriptionally inactivated in *D. simulans* and *D. mauritiana*. **(C)** Pathways for pheromone synthesis in *D. melanogaster* and *D. simulans*. 7-T is a monoene with a double bond at the 7th carbon whereas 7,11-HD is a diene with two double bonds each at the 7th and 11th carbon as the numbers in the compound names indicate.

Cuticular hydrocarbons play roles as major sex pheromones in *Drosophila* (Jallon, 1984; Yew and Chung, 2017). These compounds are poorly volatile at room temperature and thus likely to be detected by contact chemoreceptors or gustatory receptors (Kohl et al., 2015). In *D. melanogaster*, 7-tricosene (7-T) is more abundant in males than females and acts as an aphrodisiac for a female, whereas 7,11-heptacosadiene (7,11-HD) and 7,11-non-acosadiene (7,11-ND) are nearly exclusively produced by females and acts as an aphrodisiac for a male (Ferveur, 1997; Bontonou and Wicker-Thomas, 2014; Figure 1A). 7-pentacosene (7-P) is present in both sexes at lower levels also stimulates males to court (Ferveur, 1997; Bontonou and Wicker-Thomas, 2014). Conversely, 5-tricosene (5-T), 7-T and the acetylated long-chain hydrocarbon CH503 (Yew et al., 2009) present in *D. melanogaster* inhibits males from courting. Other hydrocarbons may be predominant in *Drosophila* species phylogenetically distant from *D. melanogaster* (Thompkins et al., 1993; Alves et al., 2010); in *D. virilis* females for example, 11-P and 9-T are abundant cuticular hydrocarbons (Fan et al., 2013). Aside from hydrocarbons, cis-vaccenyl acetate (cVA) produced by male ejaculatory bulb functions as a potent suppressor of male courtship (Butterworth, 1969; Antony et al., 1985; Guiraudie-Capraz et al., 2007). There is evidence that 7-T and cVA exert the courtship inhibitory effect only when these two compounds coexist (Billeter et al., 2009; Laturney and Billeter, 2016). The major source of hydrocarbon compounds is oenocytes associated with the epidermis (Ferveur, 1997; Bontonou and Wicker-Thomas, 2014), genetic ablation of which allows one to obtain flies that produce almost no hydrocarbon compounds in their cuticles (Billeter et al., 2009). Unexpectedly, such oenocyte-less flies were highly attractive as a mating partner for both females and males, implying the loss of inhibitory compounds that normally prevent indiscriminate courtship (Billeter et al., 2009). Subsequent studies identified palmitoleic acid and non-esterified versions of the fatty acid methyl esters (Dweck et al., 2015; Lin et al., 2016) as non-sex-specific attractants, potentially accounting for the sexual attractiveness found in oenocyte-less flies. The site of synthesis of these fatty acids has not been determined, but fat bodies are a likely production site (Wicker-Thomas et al., 2009; Bontonou and Wicker-Thomas, 2014; Yew and Chung, 2017). Additionally, in cactus-feeding members of the *Sophophora* subgenus (but not in the subgenus *Drosophila*), the ejaculatory bulb produce male-specific triacylglycerides (TAG) that bear combinations of branched and linear fatty acyl side chains, which act as repellents for males upon transfer to the female mate during copulation (Chin et al., 2014).

The oenocyte-less *D. melanogaster* also provided important insights into the molecular basis for species discrimination in mate choice by males: *D. melanogaster* females without oenocytes were found to provoke strong courtship even from males of other species in the *melanogaster* species subgroup (Billeter et al., 2009). Perfuming oenocyte-less *D. melanogaster* females with female-specific 7,11-HD resumed species specific courtship, i.e., attracting males of *D. melanogaster* while repelling males of other members of the *melanogaster* species subgroup (Billeter et al., 2009). In fact, unlike *D. melanogaster* females, other members of the subgroup including *D. simulans* (Figure 1A), barely produce 7,11-HD (Jallon and David, 1987). These results indicate that the contrasting preference for 7,11-HD works as an effective barrier between *D. melanogaster* and other members of the species subgroup that prevents males from engaging in interspecific courtship.

On the other hand, the opposite preference for monoenes, particularly 7-T, constitutes a mating barrier in the partial reproductive isolation between the two strains of *D. melanogaster*, i.e., African (Zimbabwe: Z) vs. cosmopolitan populations (Grillet et al., 2012). As the amount of 7-T relative to 5-T increases in courting males, cosmopolitan females become more receptive to mating, while African-Z females become less receptive (Grillet et al., 2012). These observations reinforce the view that changes in hydrocarbon compositions may be one of the key events that precede reproductive isolation between two populations under incipient speciation, providing a rationale behind the search for evolutionary changes in hydrocarbon synthesis pathways.

Several genes encoding enzymes critical for introducing a double bond have been well-characterized in *D. melanogaster*, i.e., *desaturase1* (*desat1*), *desat2*, *desatF* (also known as *Fad2*), Cyp4G1 (Qiu et al., 2012), and Bond (Ng et al., 2015). *desat1* is a pleiotropic and indispensable gene transcriptionally regulated by 5 promoters each specifying unique spatiotemporal expression (Bousquet et al., 2012): among these, promoter-*RE* functions in oenocytes and is key for pheromone synthesis (Billeter et al., 2009), while promoter-*RC* functions in neurons and is key for female receptivity (Bousquet et al., 2012; see below). *desat2* was discovered as a *desat1* homolog in the genome of an African *D. melanogaster* strain, Tai (African-T), encoding desaturase with Δ9 specificity for omega-7 hydrocarbon precursors (in contrast to desat1 with Δ7 specificity for omega-5 hydrocarbon precursors; Dallerac et al., 2000). Remarkably, *desat2* expression in African-T is female-specific, whereas *desat2* is not expressed at all in the cosmopolitan Canton-special (CS) strain due to a promoter defect. Nucleotide sequence comparisons suggest that the *desat2* gene structure in cosmopolitan populations is a descendant of that in the African counterpart (Takahashi et al., 2001). The presence or absence of functional *desat2* in the African and cosmopolitan *D. melanogaster* nicely explains why females of African *D. melanogaster* preferentially produce 5,9-HD (an omega-7 hydrocarbon) rather than 7,11-HD (an omega-5 hydrocarbon), the latter of which dominates in females of cosmopolitan *D. melanogaster* instead. In contrast, the different cuticular contents of 5-T and 7-T in males from the two populations have been demonstrated to be an important factor for females in choosing a mate, as discussed above. However, the different 5-T vs. 7-T ratio in African and cosmopolitan males cannot be ascribable to the presence or absence of functional *desat2* in the respective genomes, because males do not exhibit *desat2* expression in both populations. Thus, the significance of the discovered genomic changes in the *desat2* gene in incipient speciation in *D. melanogaster* populations has not been fully validated.

*desatF* was identified as the gene that plays a central role in the synthesis of 7,11-HD and other dienes with two double bonds that are predominant in *D. melanogaster* females (Chertemps et al., 2006): DesatF catalyzes the reaction to introduce the second double bond into fatty acid precursors (Figures 1B,C). It was shown that female-specific *desatF* expression relies on a female-determinant, Transformer (Tra), and *desatF* knockdown in females results in a dramatic increase in monoenes (e.g., 7-T) at the expense of dienes (e.g., 7,11-HD). Comprehensive species comparisons of the *desatF* structure and expression unraveled the exceedingly complex evolutionary changes this gene underwent (Shirangi et al., 2009). Although a conserved *desatF* sequence is recognizable in the genomes of 18 out of 24 species examined, it is not functional in 9 species: the *desatF* gene is translationally inactive in 6 species (although *desatF* in some species retains an intact open reading frame, ORF) and it harbors mutations in the coding sequence in 3 species (Shirangi et al., 2009). The *desatF* gene in some species underwent multiple transitions, e.g., once transcriptionally inactivated, it was transcriptionally reactivated and ultimately ORF-disrupted (Shirangi et al., 2009). Remarkably, female-specific expression as in *D. melanogaster* is not conserved across species that carry an active *desatF* gene: among the species examined, *D. sechellia*, *D. errecta*, and *D. melanogaster* are the only ones that exhibit *desatF* sexually dimorphic expression. The transitions between the monomorphic and dimorphic expression were found to be associated with the loss and gain of distinct biding sites for the sex-determinant transcription factor Doublesex (Dsx) in the *cis*-regulatory region of the *desatF* gene, respectively (Shirangi et al., 2009). In the *D. melanogaster* species subgroup, a common ancestor presumably had dimorphic expression of *desatF* and thus expressed 7,11-HD, which was subsequently lost as a result of transcriptional inactivation of *desatF* in the clade containing *D. simulans* and *D. mauritiana*, while dimorphic expression was sustained in the clade to *D. melanogaster* (Figure 1B). It is thus plausible that reproductive isolation between the two sympatric sibling species *D. melanogaster* and *D. simulans* was endowed, in part, by *cis* element mutations in the *desatF* gene, which removed 7,11-HD from females of *D. simulans*, in concordance with changes in the preference for 7,11-HD in males (see below).

Yet another gene of interest is *fatty acid elongase F* (*eloF*), which elongates the DesatF products omega-7,11 fatty acids, the precursors of 7,11-HD and 7,11-ND in *D. melanogaster* females (Chertemps et al., 2007; Figure 1C). *eloF* is expressed in a female-biased manner in *D. melanogaster* (Chertemps et al., 2007) and *D. sechellia* (Combs et al., 2018) and is not expressed at all in *D. simulans* (Chertemps et al., 2007; Figure 1A). What we see here with *eloF* is exactly the above-described pattern of *desatF* expression in these three species. It remains an open question whether this kind of coordinated evolution of *eloF* and *desatF* can be generalized into other clades of the *Drosophila* phylogeny.

cVA is probably the most studied among pheromones in *Drosophila*, but little is known about its biosynthesis. Unlike major cuticular hydrocarbon pheromones that are produced by oenocytes, cVA is secreted into the lumens of ejaculatory bulb in a male and ejected, together with sperms, into the female genitalia during copulation, reducing the sexual attractiveness of that female for other males (Antony et al., 1985). In addition to such an anti-aphrodisiac effect, cVA enhances aggression among unfamiliar males (Wang and Anderson, 2010; Wang et al., 2011) but reduces aggression among familiar males (Liu et al., 2011), and promotes non-sex-specific aggregation in a context-dependent manner (Bartelt et al., 1985; Wertheim et al., 2002; Lebreton et al., 2014; Cazalé-Debat et al., 2019), and suppresses male courtship toward a virgin female after his exposure to a mated female (Ejima et al., 2007; Keleman et al., 2012). Radioactive tracer labeling of metabolites supported the hypothesis that the male ejaculatory bulb synthesizes cVA from acetate as a starting compound, yet the vaccenyl moiety is of an unknown origin (Guiraudie-Capraz et al., 2007). Notably, in *D. buzzatii*, radiolabeled acetate similarly incubated with male ejaculatory bulb yields two ketone compounds, i.e., (Z)-10-heptadecen-2-one, an aggregation pheromone, and its antagonist, 2-tridecenone (Skiba and Jackson, 1993). A large number of long-chain acetates, alcohols and ketones have been reported as aggregation pheromones in *Drosophila*, and the composition of pheromone blends varies widely across species (Symonds and Wertheim, 2005; Lebreton et al., 2017). It remains to be determined whether these aggregation pheromones also play roles as sex pheromones, and if so, how significant they are in reproductive isolation in speciation events.

## Sex Pheromone Reception

In the previous section, we saw that a single pheromone may exert contrasting reactions in different species. A favored interpretation for this would be that a receptor for the pheromone responds differently in different species. In this section, we review our current understanding of contact chemoreceptors for pheromones in *Drosophila* and evaluate the above hypothesis.

**FIGURE 2:**
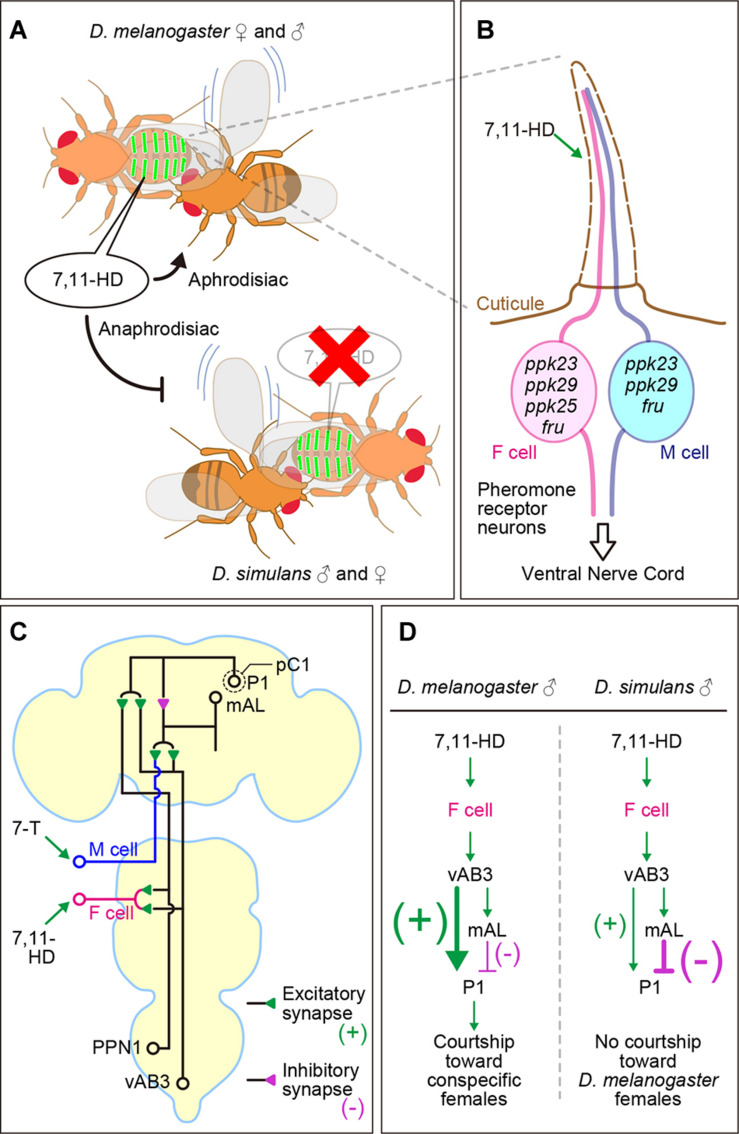
*D. melanogaster* males are attracted and *D. simulans* males are repelled by 7,11-HD. **(A)** Contrasting responses to 7,11-HD underlie conspecific mate choice. **(B)** F-cell and M-cell in the male tarsi sense female pheromones (e.g., 7,11-HD) and male pheromones (e.g., 7-T), respectively. The F-cell and M-cell both express *ppk23*, *ppk29* and *fru*, while *ppk25* expression is F-cell specific. **(C)** Central pathway for 7,11-HD perception in male flies involves ascending excitatory (+) neurons including vAB3 and PPN1, mAL inhibitory (–) interneurons, and courtship triggering P1 excitatory (+) interneurons. **(D)** mAL-mediated inhibition overwhelms vAB3-mediated excitation in P1 neurons in *D. simulans* males but not *D. melanogaster* males, resulting in opposite responses to 7,11-HD in males of these two species. P1 represents a male-specific subset in the pC1 neuron group (circled by a dotted line). Circles, lines, and triangles indicate somata, neurites, and presynaptic terminals of neurons, respectively.

Electrical recordings of receptor potentials and spiking activities from a receptor cell are the straightforward functional demonstration of ligand-receptor interactions. The female pheromone 7,11-HD was demonstrated to provoke discharges from chemosensory neurons in the foreleg tarsi of *D. melanogaster* males (Toda et al., 2012; Figure 2A). When a male fly taps the female abdomen with his foreleg during courtship, these chemosensory neurons will be stimulated by cuticular hydrocarbon compounds on the female abdomen. A fraction of the foreleg chemosensory neurons express the neural masculinizing protein Fruitless (FruM), and these *fru*[+] chemosensory neurons exhibit sex differences in the central projection pattern (Kimura et al., 2019; see below). A subset of such *fru*[+] chemosensory neurons in foreleg tarsi express *ppk23* and related genes that encode Degenerin/Epithelial Na^+^ channel (Deg/ENaC) family proteins, which have been implicated in 7,11-HD-dependent male courtship based on behavioral phenotypes upon targeted knockdown and Ca^2+^ neural activity imaging (Liu et al., 2012; Lu et al., 2012; Thistle et al., 2012; Vijayan et al., 2014; see below; Figure 2B).

The identity of 7,11-HD responsive cells was further defined by Ca^2+^ activity imaging: the relevant tarsal sensillum houses a pair of *fru*[+]/*ppk23*[+]/*ppk29*[+] cells, each having a complementary function such that one (the F-cell) responds to the female pheromone 7,11-HD but not the male pheromone 7-tricosense (7-T), while the other (the M-cell) responds to 7-T but not 7,11-HD (Thistle et al., 2012; Figure 2B). Up- and down-regulation of F-cells promote and repress male courtship activities, respectively, and the converse effects are observed when M-cells are similarly manipulated (Lu et al., 2012; Starostina et al., 2012; Thistle et al., 2012; Toda et al., 2012). The F-cells are molecularly distinguishable from the M-cells by their expression of *ppk25*, which is required specifically for the 7,11-HD responses of these cells (Vijayan et al., 2014; Figure 2B). The F-cells on foreleg tarsi are probably the major sensor for 7,11-HD, although there are other cells that are thought to be additional 7,11-HD sensors (see below). The F-cells and M-cells are present in both sexes, and their sex-specific functions are encoded by sex-specific functions via sexually dimorphic projections (Thistle et al., 2012).

7-T may stimulate additional cells other than the M-cells in the tarsus, including *Gr32a*-expressing bitter responsive cells (Koganezawa et al., 2010; Wang and Anderson, 2010) that are negative for both *ppk23* (Thistle et al., 2012) and *fru* (Koganezawa et al., 2010) expression; *ppk23*-positive cells are located predominantly in the ventral sensory hairs, whereas *Gr32a*-positive cells are located mostly in the dorsal sensory hairs (Ling et al., 2014). Furthermore, Lacaille et al. (2007) showed, using a tungsten electrode inserted into the base of a sensillum, that some bitter-sensitive cells on a labial palp (i.e., mouth) contained sensory neurons responsive to low concentrations of 7-T, a pheromone that inhibits male courtship. Because male flies lick female genitalia during courtship, sensory neurons on the labial palp are likely activated in courting males.

7-T is not the sole ligand for the M-cells: cVA also activates these cells (Thistle et al., 2012). This is rather surprising, because cVA is volatile and known to activate primarily the olfactory receptor neurons expressing *Or67d* (Ha and Smith, 2006; Kurtovic et al., 2007; Datta et al., 2008; Ruta et al., 2010; Liu et al., 2011) and secondarily those expressing *Or65a* (Ejima et al., 2007; Liu et al., 2011; Lebreton et al., 2014) in the antenna. In addition to these tarsal sensory cells, a subset of gustatory cells respond to fly cuticle extracts and promote mating activities in both female and male *D. melanogaster* (Koh et al., 2014; He et al., 2019).

What are the roles of these contact chemosensory cells in sexual isolation among *Drosophila* species? Fan et al. (2013) showed that RNAi-mediated knockdown or genetic ablation of *Gr32a*-expressing neurons in *D. melanogaster* males restores the attractiveness of oenocyte-less *D. melanogaster* females that were perfumed with cuticular extracts from females of other *Drosophila* species (i.e., *D. simulans*, *D. yakuba*, or *D. virilis*) or with synthetic 7-T, 9-T and/or 11-P, the treatments that otherwise abrogate the sex appeal of *D. melanogaster* females (Fan et al., 2013). These results suggest that *Gr32a*-expressing sensory neurons that are responsive to a broad spectrum of hydrocarbons play a key role in the conspecific preference in *D melanogaster* males. Subsequently, similar behavioral assays were conducted with *Gr32a*-knockout *D. simulans*, which was generated by CRISPR/Cas9-mediated targeted mutagenesis, yielding a contrasting result: *Gr32a* mutant males of *D. simulans* displayed no sign of impairment in discriminating conspecifics from other species, exhibiting a strict preference for females of the same species (Seeholzer et al., 2018; Ahmed et al., 2019). The lack of effect of *Gr32a* knockdown on mating discrimination is intriguing in view of the fact that not only *Gr32a* expression in tarsal sensory neurons but also the function of *Gr32a* in bitterness perception were conserved between *D. melanogaster* and *D. simulans* (Ahmed et al., 2019). *ppk25* knockout in *D. simulans*, on the other hand, diminished male courtship activities toward conspecific females, as in *D. melanogaster* (Ahmed et al., 2019). However, the primary stimulant of *ppk25*-expressing tarsal sensory neurons in *D. melanogaster* is 7,11-HD, which repels *D. simulans* males, implying that *ppk25*-expressing tarsal neurons in *D. simulans* promote male courtship when activated by a pheromone other than 7,11-HD. Alternatively, the pathway initiated by the *ppk25*-expressing sensory neurons is not a simple accelerator of male courtship activity; instead, inputs through this pathway may gain either positive or negative valence upon central integration, which varies depending on the species and context (Figures 2C,D). It is an interesting question as to which mechanisms—the peripheral or central mechanisms—are more frequently modified for sexual isolation in incipient speciation.

## Molecular Identity of Contact-Chemical Pheromone Receptors

It remains an open question as to which proteins function as specific receptors for pheromones. As described above, the reception of major contact-chemosensory pheromones is mediated by cells that express select *ppk* family members (e.g., *ppk23*, *ppk25*, and *ppk29*) or *Gr32a*. *Gr68a* (Bray and Amrein, 2003) and *Gr33a* (Watanabe et al., 2011) have also been suggested to have roles in mating behavior. More recent works have further shown that a subset of the ionotropic glutamate receptor (IR) family contributes to courtship behavior (Koh et al., 2014; He et al., 2019). Are these proteins by themselves function as receptors for pheromones? Are they required for signal transduction downstream of receptors? Liu et al. (2020) argue that *ppk23*, *ppk25*, and *ppk29* form a functional receptor for 7,11-HD based on the observation that the otherwise 7,11-HD-unresponsive M-cells acquire sensitivity to this compound when the *ppk* trio is expressed in the cells. It should be noted that only *ppk25* needs to be overexpressed because *ppk23* and *ppk29* are endogenously expressed in the M-cells. This finding in the M-cells is in line with the aforementioned result in the F-cells that *ppk25* knockdown abrogates their sensitivity to 7,11-HD, which resumes upon *ppk25* overexpression (Vijayan et al., 2014). Nonetheless, these observations do not exclude the possibility that the *ppk* proteins are not receptors that bind 7,11-HD but rather are their effector channels for electrogenesis, amplifying signaling downstream of the receptors (Ng et al., 2019). *ppk25* overexpression might have enhanced the outputs of the receptors that intrinsically respond to a wide spectrum of agonists so that even small responses that might otherwise be overlooked become detectable by the experimental manipulation.

**FIGURE 3:**
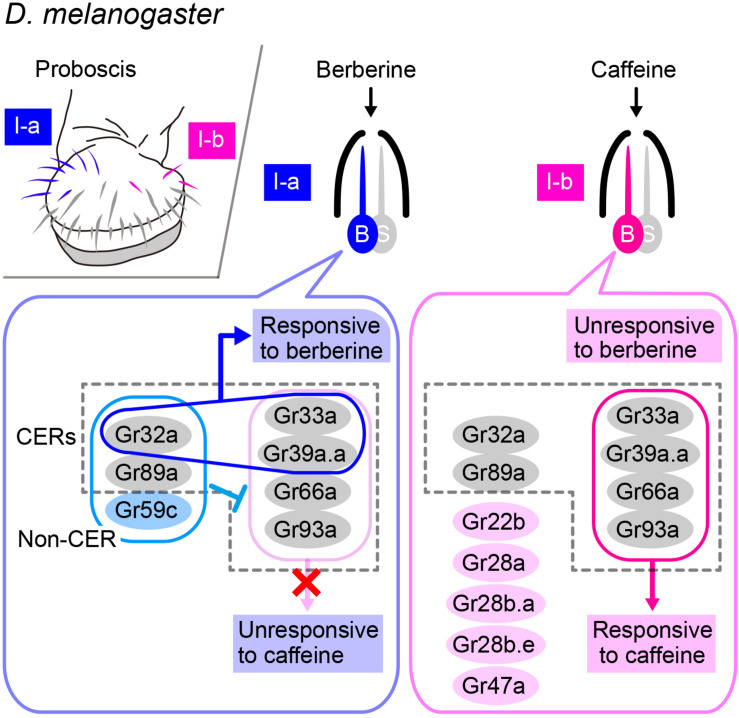
Combination of several GRs expressed in a single neuron determines the response spectrum of the cell. Upper left-side panel: Spatial localization of bitter-responsive I-a (blue) and I-b (red) sensilla on the labellum. Right-side panels: The bitter-responsive neuron (labeled as “B”) of each sensillum expresses a different combination of Gustatory receptors (Grs), five of which are referred to as “Commonly Expressed Receptors” (CERs; in dotted rectangles) and are expressed in every bitter-responsive neuron on the labellum. Expression of Gr32a, Gr33a, and Gr39a.a together confers a berberine sensitivity on the I-a sensillum, whereas expression of Gr33a, Gr39a.a, Gr66a, and Gr93a together confers a caffeine sensitivity on the I-b sensillum. However, the latter four Grs are unable to confer a caffeine sensitivity on the I-a sensillum, because Gr32a, Gr59b, and Gr89a are coexpressed in this sensillum.

Gr32a is another candidate receptor for hydrocarbon pheromones, particularly 7-T. Gr32a belongs to the insect chemoreceptor superfamily, which is composed of 68 Grs and 62 Ors, which are 7-pass transmembrane proteins that form ion channels on their own without any involvement of additional cytoplasmic factors (i.e., ionotropic receptors), unlike mammalian chemoreceptors, which are typically 7-pass membrane proteins with inverse topology (in comparison with that of insect receptors) that act via a G-protein mediated transduction cascade (i.e., metabotropic receptors; Sato et al., 2008, 2011). A recent cryogenic electron microscopy (cryoEM) study on an Or—namely, the odorant receptor co-receptor (Orco)—in an insect identified a crevice of 10-Å depth and 20 Å length within the extracellular leaflet, along which several residues known to affect ligand sensitivity lie, and which is thus likely to serve as a binding site for ligands (Butterwick et al., 2018). Following the analogy of Orco, Gr32a may have ligand binding activity. However, Gr32a is widely expressed in contact-chemosensory neurons that respond to a wide spectrum of ligands, particularly those known as bitter tastants, raising the question of how the Gr32a protein confers the ligand specificity on the sensory neurons. A recent exhaustive analysis of ligand-receptor-neuron relationships for gustatory responses in the labial palp defined Gr32a, Gr33a, Gr39a.a, Gr66a, Gr89a, and Gr93a as commonly expressed receptors (CERs) in bitter-sensitive receptors, which are equivalent to Orco in olfactory receptors (Dweck and Carlson, 2020; Figure 3). Typically, two, three, or four fixed members of CERs need to be coexpressed for normal bitter sensitivity: Gr32a, Gr33a, and Gr66a are the triple constituents and Gr33a, Gr39a, Gr66a, and Gr93a are the quadruple constituents essential for responding to caffeine and some other compounds in a subset of bitter-sensitive chemosensory neurons housed in I-a and I-b sensilla, respectively. When one component of the trio or quartet is lost, the neurons may simply become unresponsive to nearly all bitter tastants to which they normally respond or, alternatively, the neurons may acquire a novel ligand selectivity depending on their neuron type, which would suggest competition among multiple Gr species expressed in the same neuron in forming a functional heteromeric receptor for bitter tastants (Dweck and Carlson, 2020). Thus, the response spectrum of a neuron may change dependent on the combination of Gr species coexpressed and the relative abundance of different Grs. These considerations tempted us to suggest that Gr32a may contribute to the reception of 7-T and other pheromones as one of the CERs.

Then, the question remains as to how the response spectrum of a contact-chemosensory neuron is specified. The I-a and I-b sensilla are morphologically similar, but each respond to mutually exclusive sets of bitter compounds in *D. melanogaster*: for instance, caffeine elicits responses from I-b but not I-a, whereas berberine elicits responses from I-a but not I-b. However, the I-a sensillum acquires the entire response spectrum of I-b and thus becomes responsive to caffeine when the non-CER Gr59c is lost or when one CER, either Gr32a or Gr89a, is lost (Dweck and Carlson, 2020). Conversely, misexpression of Gr59c in the I-b sensillum confers the I-a type response spectrum on the I-b sensillum, provided that Gr32a and Gr89a are intact (Dweck and Carlson, 2020). This and other experiments demonstrate that the I-a sensillum does not respond to caffeine and other ligands that normally activate the I-b sensillum, because Gr59c in addition to the CER Gr32a and Gr89a coordinately suppress the responses to these substances (Dweck and Carlson, 2020). These observations imply that evolutionary loss or gain of the expression of just one of the Gr-coding genes could produce substantial changes in the ligand specificity of a subset of contact-chemosensory neurons, thereby leading to diversified pheromonal responses that potentially impact speciation events.

## Central Processing of Contact-Chemical Pheromone Information in Evolution

Contact chemosensory information plays a pivotal role in recognizing potential mating partners, making a decision to court, and initiating the mating motor program in *Drosophila*. One pheromone substance may induce different behavioral responses in recipients of different species, with the differences potentially arising from the different response properties of peripheral receptor cells as discussed above or the different processing of pheromone inputs in the central nervous system (CNS). In this section, we focus on central mechanisms underlying the different behavioral responses to contact chemosensory pheromones among *Drosophila* species in the context of mate preference. Unfortunately, there are no means for systematic labeling of a single neuron along the entity of the cell in non-model species, hampering circuit dissection in these species. Due to this technical difficulty, species differences in the structure and function of central neurons have been least explored. One exception is a study which successfully unraveled the central circuit basis for the biased preference of conspecific over sibling species females by *D. simulans* males (Seeholzer et al., 2018).

The central circuit for mating behavior has been extensively analyzed in *D. melanogaster* (Kohl et al., 2013; Yamamoto and Koganezawa, 2013), in which FruM-positive neurons tend to interconnect in forming the core portion of the circuit (Ruta et al., 2010; Yu et al., 2010). A male-specific interneuron group called P1 (Kimura et al., 2008) or its subpopulation (Ishii et al., 2020) plays a decisive role in initiating courtship behavior (Yamamoto and Koganezawa, 2013; Figure 2C). The P1 neuron cluster was first identified as a subset of FruM/Dsx double-positive neurons (20 neurons per hemisphere) that could drive test females to perform male-type courtship behavior toward a target female when those neurons were clonally masculinized in the test female brain by the *tra*^1^ mutation (Kimura et al., 2008); the *tra*^1^ mutation removes the DsxF feminizer protein that otherwise kills P1 precursor cells during development, thereby allowing male-specific P1 to persist throughout the adult stage in the female brain (Kimura et al., 2008; see also Ren et al., 2016). In a solitary male, artificial activation of P1 neurons via heat-sensitive dTrpA1 channels or the light-activatable channel Channelrhodopsin induces the early steps of courtship, i.e., unilateral wing extension and vibration for singing and tapping with forelegs (Kohatsu et al., 2011; Kohatsu and Yamamoto, 2015). Ca^2+^ imaging of P1 neurons in a tethered male on a treadmill revealed that these neurons are excited when the male touches the female abdomen with his foreleg (Kohatsu et al., 2011). P1 neurons remain continuously and dynamically active throughout the courtship achievements under freely moving (Grover et al., 2016, 2020) as well as tethered (Kohatsu and Yamamoto, 2015) conditions. Contact chemosensory sensation of female cues is crucial for courtship initiation by the male, based on the finding that touch-induced chasing is blocked when the virgin female as a courtship target is perfumed with the hexane extract of male cuticles (Kohatsu et al., 2011). P1 neurons have been shown to be excited when the male foreleg tarsus is touched by a glass rod, provided that it is coated with the hexane-extract of fly cuticles (Kohatsu et al., 2011). Notably, P1 neurons exhibit Ca^2+^ rises upon tarsal stimulation with the extracts of male as well as female cuticles, although female extracts provoke significantly larger responses than male extracts do (Kohatsu et al., 2011). These and other observations support the notion that P1 neurons in the male brain receive contact-chemosensory inputs originating from tarsal pheromone receptors upon the touch of a female and drive persistent courtship toward the female. P1 neuron outputs are relayed by descending interneurons that activate the motor pattern generator for courtship acts (Clyne and Miesenböck, 2008; Kohatsu et al., 2011; von Philipsborn et al., 2011; Kimura et al., 2015; Cande et al., 2018; Clemens et al., 2018; Namiki et al., 2018; McKellar et al., 2019). A subset of P1 neurons provoke not only courtship toward a female but also aggression toward a male (Inagaki et al., 2014; Hoopfer et al., 2015; Koganezawa et al., 2016), presumably dependent on the sensory inputs they receive (Ishii et al., 2020; Wohl et al., 2020), while inhibiting sleep in a manner dependent on the internal state of the fly (Chen et al., 2017; Wu et al., 2019). Note, however, that subpopulations of the P1 cluster and the pC1 cluster to which the P1 cluster belongs need further clarification in terms of functional specialization (see Costa et al., 2016). The internal states, such as the motivational state and sleep/arousal cycle, affect P1 activities via dopaminergic and GABAergic synaptic inputs to promote and inhibit courtship, respectively (Crickmore and Vosshall, 2013; Zhang et al., 2016, 2018).

Given that P1 neurons in the brain trigger the lower center that produce motor outputs for courtship actions, how does the pheromone information received by sensory cells in the legs and mouth reach to the P1 neurons? The pathways through which contact chemical pheromone inputs reach to P1 neurons were revealed in *D. melanogaster* by anatomical detection of putative synaptic contacts in conjunction with Ca^2+^ imaging to monitor the neural activities across synapses (Figure 2C). The majority of the *ppk23*-positive tarsal chemosensory neurons responsive to 7,11-HD appear to terminate their axons in the prothoracic ganglion (Mellert et al., 2010; Kimura et al., 2019), and thus direct contact with brain-intrinsic P1 neurons must, if any, be limited. Instead, the ascending interneuron group vAB3 intervene in the communication between the *ppk23*-positive sensory neurons and P1 neurons: vAB3 neurons originate in the abdominal ganglion and terminally project to the lateral protocerebrum, the brain region P1 neurons densely innervate, with *en passant* arbors in the prothoracic and suboesophageal ganglia (Clowney et al., 2015). vAB3 neurons are excited when the male touches the female abdomen with his foreleg, and vAB3 activation by Ach iontophoretically applied to the prothoracic neuropil induces Ca^2+^ elevation in P1 neurons, which is blocked by vAB3 severing (Seeholzer et al., 2018). Thus, vAB3 provides an excitatory pathway that conveys the female pheromone information from leg sensory neurons to P1 neurons that initiate male courtship. Another group of neurons that are likely presynaptic to P1 are the mAL neurons, which are *fru*-positive GABAergic inhibitory interneurons (Koganezawa et al., 2016) that are sexually dimorphic in both structure and cell number (Kimura et al., 2005). A sexually dimorphic neurite of mAL likely contacts, in a male-specific manner, the axon terminals of *Gr32a-*expressing tarsal sensory neurons (Koganezawa et al., 2010). Remarkably, mAL neurons exhibit Ca^2+^ elevation in response to activation of vAB3, whose *en passant* arbors appear to intermingle with mAL arbors in the suboesophageal ganglion. This observation raises the possibility that vAB3 could also deliver an inhibitory input to P1 neurons via mAL neurons. Indeed, P1 activation in response to stimulation of vAB3 is significantly greater after mAL severing, supporting the notion that the reception of aphrodisiac female pheromones by the leg chemosensory receptors ultimately provokes not only excitatory responses but also inhibitory responses in P1 neurons, the decision-making neural center for male courtship behavior. Convergence of these two antagonistic inputs at nearly the same time might create a sensitized condition where additional cues easily bias the activity of P1 neurons that are involved in decision-making to court or not, allowing the male fly to judge whether the confronting target for courtship is truly an appropriate potential mate. There is yet another ascending interneuron group, PPN1, that convey inputs originating from *ppk23/ppk25* double-positive pheromone receptors (female-pheromone sensitive F-cells) to P1 neurons; PPN1 neurons act as excitatory presynaptic fibers for P1 and, at the same time, act as an element in the inhibitory pathway impinging on P1 via mAL interneurons (Kallman et al., 2015). In contrast to F-cell axons, which terminate mostly in the thoracic ganglia, a subset of *ppk23*-positive and *ppk25*-negative M-cells extend their axons beyond the thorax and terminate in the suboesophageal ganglion, where these axons seem to come into contact with an mAL neurite (Kallman et al., 2015). As a consequence, the M-cell activator 7-T primarily inhibits P1 neuron activity and thus represses male courtship, whereas the F-cell activator 7,11-HD elevates P1 neuron activity despite its inhibitory effect through mAL and ultimately promotes male courtship (Kallman et al., 2015). Thus, we find that a common excitatory pheromone input is fed into two pathways, one converts the excitatory signal into an inhibitory signal, while the other conveys the excitatory signal without inverting its sign, and the two pathways ultimately converge onto the P1 neurons.

This principle would offer a simple means to fine-tune the sensitivity of a decision-making neural center to incoming sensory cues. In fact, different preferences for 7,11-HD in males of the *D. melanogaster* species subgroup are suggested to involve a shift in the excitatory vs. inhibitory balance in contact chemosensory inputs impinging on P1 neurons (Figure 2D). As discussed in the preceding sections, males of *D. simulans* avoid 7,11-HD, which is specifically enriched in female cuticles of *D. melanogaster*. The neural pathway through which 7,11-HD-induced activities travel to P1 neurons is, in principle, conserved between *D. melanogaster* and *D. simulans*. As in *D. melanogaster*, *D. simulans ppk23*-positive sensory neurons activate vAB3 ascending interneurons, which in turn produce activities in mAL inhibitory interneurons (Seeholzer et al., 2018). Notably, P1 neurons exhibit no apparent activation when vAB3 is depolarized in *D. simulans*. Upon mAL severing, however, vAB3 activation induces noticeable Ca^2+^ rises in P1 neurons (Seeholzer et al., 2018). These observations suggest that both direct excitatory and indirect inhibitory connections between vAB3 and P1 also exist in *D. simulans*, but in the latter species inhibitory inputs overwhelm excitatory inputs, and, as a consequence, 7,11-HD is unable to activate P1 and thus unable to trigger male courtship behavior in *D. simulans* (Seeholzer et al., 2018; Figure 2D). This species difference in the integrative functions of the CNS circuit represents a plausible mechanism for the premating isolation between *D. melanogaster* and *D. simulans*, which involves contrasting preferences for 7,11-HD: attraction in *D. melanogaster* males and avoidance in *D. simulans* males. An intriguing evolutionary scenario is that selective pressure acted on synapses associated with male-specific P1 neurons to shift the balance in favor of excitatory inputs from vAB3 against inhibitory inputs from mAL in an ancestral species of *D. melanogaster*, when females of this species acquired some dienes as new pheromone components, including 7,11-HD on their cuticles. The postulated shift in the balance between excitatory and inhibitory synaptic efficacies needs to be experimentally demonstrated. Also, if a species difference in the synaptic efficacy exists, as expected, it remains to be determined what genetic change is responsible.

## Crosstalk Between Contact-Chemosensory and Olfactory Pathways

In this article, we focused on the contact-chemosensory signaling that plays a key role in mate choice across *Drosophila* species. However, other sensory modalities also have substantial impacts on partner preference in these flies (Krstic et al., 2009) and the relative contributions of different modalities to mating vary from species to species (Spieth, 1952). Studies in *D. melanogaster* revealed that males rely primarily on visual (Pan et al., 2012; Kohatsu and Yamamoto, 2015) and auditory (von Schilcher, 1976; Ishikawa et al., 2019) cues in tracking a courtship target, while chemosensory cues play major roles in triggering and maintaining courtship actions. In contrast to chemosensory inputs that impinge onto the courtship decision-making P1 neurons (Kohatsu et al., 2011; Clowney et al., 2015; Kallman et al., 2015), auditory and visual inputs seem to be processed by interneurons distinct from P1 neurons, respectively (Ribeiro et al., 2018; Deutsch et al., 2019). It remains to be clarified how the visual and auditory information is integrated with the P1-dependent command in driving courtship behavior.

In many other insects, volatile compounds are commonly used as pheromones, which are processed by olfactory channels in recipient animals and elicit long distance attraction or avoidance (Fleischer and Krieger, 2018). The best-characterized volatile pheromone in *Drosophila* is cVA, which acts through both olfactory and contact-chemosensory pathways (Thistle et al., 2012; Ejima, 2015), and thus these two modalities in fact interact to affect fly mating behavior. Crosstalk between the contact-chemosensory and olfactory systems in controlling mating and other behaviors is probably prevalent (Wang et al., 2011; Laturney and Billeter, 2016), partly reflecting the fact that the same pheromone compound can exist in either the solid/liquid or vapor state at temperatures a fly engages in reproduction. Of note, 7,11-HD is a precursor of Z-4-undecanal, which is known to function as a long range, species-specific, aggregation pheromone detected by odorant receptor Or69a (Lebreton et al., 2017). Alternatively, it might be that Grs can detect volatile compounds and Ors can detect non-volatile compounds.

Crosstalk between contact chemosensory and olfactory pathways also underlies courtship enhancement by food odor. Phenylacetic acid and phenylacetaldehyde are aromatic odors associated with fruit and other plant tissues that feed *Drosophila* flies and provide oviposition sites. These compounds are received by IR84a- and *fru*-expressing olfactory receptor neurons (ORNs) in the antenna. The projection neurons postsynaptic to IR84a ORNs extend their axons into the pheromone-specialist fiber tract even though they convey food odor information (Bates et al., 2020). As a consequence, IR84a-mediated food odor information is sent to a pheromone processing region of the lateral horn, where it is probably integrated together with pheromone information to control mating behavior (Grosjean et al., 2011). Conversely, male-specific cuticular hydrocarbons or cVA deposited onto food promotes landing responses in flying female and male flies, although the neural basis for this effect is not known (Cazalé-Debat et al., 2019; see also Lin et al., 2015; Dumenil et al., 2016). Therefore, crosstalk between contact-chemosensory and olfactory information takes place in both the peripheral and central neural circuitries, and the modes of crosstalk appear to be built in a hardwired connectivity blueprint. Which neurons in the mating circuit receive and process inputs from food-odor interneurons remain unknown. In view of the highly variable feeding habits across species, the circuit bases involved in the integration of food odor and mating signals would also be diversified across species.

A recent comparative study on the olfactory basis for hostplant preference in the *Drosophila melanogaster* subgroup unraveled multilayered modifications at different nodes of olfactory information processing (Auer et al., 2020). *Drosophila sechellia* is a monophagous species specifically associated with noni fruit (*Morinda citrifolia*), whereas the sibling species *D. simulans* is polyphagous, as are several other members of the group. Long distance attraction to noni fruit in *D. sechellia* depends on at least three modifications of the common design for the olfactory circuitry: specialization in the response spectrum of the olfactory receptor Or22a, an increase in the number of Or22a-harboring sensilla, and acquisition of novel terminal arbors in the lateral horn by the DM2 projection neurons that are postsynaptic to Or22a ORNs (Auer et al., 2020). Yet another study suggested that the odorant binding protein genes *Obp57d* and *Obp57e* were specialized in *D. sechellia* to make this species prefer noni fruit odor, whereas these genes are required for avoiding noni fruit in the sibling species *D. simulans* (Matsuo et al., 2007). This study used species hybrids in conducting unbiased screens for genetic loci that are decisive in contrasting noni fruit preferences between *D. sechellia* and *D. simulans*. A similar and even more thorough approach with species hybrids would be fruitful in identifying a collection of genes that are required for diversified mate preferences.

## Perspectives

The neural mechanism for mating behavior could have accumulated a variety of changes at multiple circuit nodes within the homologous neural pathways across different phylogenetic lineages. Among the members of the *D. melanogaster* species subgroup, species hybrids are relatively easy to obtain, and would offer an ideal platform for studying genome-wide identifications for loci responsible for species differences in mate preference (Castillo and Barbash, 2017). Indeed, genotype-phenotype correlative analyses with whole genome sequencing and behavioral phenotype classification for every hybrid fly is now feasible. Subsequent CRISPR/Cas9-targeted mutagenesis in conjunction with *piggyBac*-based transgenic rescue will be used to assure the causality between the gene and behavior (Tanaka et al., 2016, 2017). The entire brain connectome is near completion in *D. melanogaster*, providing a solid reference map of brain circuitries for the study of neuroanatomy in other members of the *D. melanogaster* species subgroup. We may soon witness the beginning of a new era in the history of evolutionary studies of the neural basis of reproductive isolation and behavioral divergence.

## Author Contributions

DY: conceptualization, review, and editing. KS and DY: funding acquisition and writing the original draft. Both authors contributed to the article and approved the submitted version.

## Conflict of Interest

The authors declare that the research was conducted in the absence of any commercial or financial relationships that could be construed as a potential conflict of interest.
